# Augmented Visual Feedback: Cure or Distraction?

**DOI:** 10.1177/0018720820924602

**Published:** 2020-06-03

**Authors:** Yke Bauke Eisma, Clark Borst, René van Paassen, Joost de Winter

**Affiliations:** 1 2860 Delft University of Technology, The Netherlands

**Keywords:** visual attention, eye-tracking, human–machine interfaces

## Abstract

**Objective:**

The aim of the study was to investigate the effect of augmented feedback on participants’ workload, performance, and distribution of visual attention.

**Background:**

An important question in human–machine interface design is whether the operator should be provided with direct solutions. We focused on the solution space diagram (SSD), a type of augmented feedback that shows directly whether two aircraft are on conflicting trajectories.

**Method:**

One group of novices (*n* = 13) completed conflict detection tasks with SSD, whereas a second group (*n* = 11) performed the same tasks without SSD. Eye-tracking was used to measure visual attention distribution.

**Results:**

The mean self-reported task difficulty was substantially lower for the SSD group compared to the No-SSD group. The SSD group had a better conflict detection rate than the No-SSD group, whereas false-positive rates were equivalent. High false-positive rates for some scenarios were attributed to participants who misunderstood the SSD. Compared to the No-SSD group, the SSD group spent a large proportion of their time looking at the SSD aircraft while looking less at other areas of interest.

**Conclusion:**

Augmented feedback makes the task subjectively easier but has side effects related to visual tunneling and misunderstanding.

**Application:**

Caution should be exercised when human operators are expected to reproduce task solutions that are provided by augmented visual feedback.

## Introduction

Automation is present in many aspects of society, including areas such as process control, human transportation (e.g., driverless metro trains), and warehouse logistics. However, in complex work domains such as air traffic control (ATC), anesthesia care, and car driving, full automation is not yet feasible because of the high risks involved (Bazilinskyy et al., 2019; Kaber & Endsley, 2004; Parasuraman et al., 2000). Although information acquisition and analysis are highly automated, final decision making is left to a human operator. In ATC, for example, a human controller supervises radar screens to decide which routing instructions to give to pilots in order to structure the airflow safely and efficiently (Sheridan, 2002).

A crucial question for the above domains is what information should be shown on the display and what visual appearance the information should have. One approach would be to present all the data that the operator might need. However, as explained by Sheridan (1995), “humans can absorb and make use of only very limited quantities of information. It is well established that displaying all the information that might be useful means there is too much information to be able to find what is needed” (p. 825). Another approach, which is the focus of the current paper, would be to let the computer transform the available sensor data into intuitive visualizations for the task at hand. This approach may be attractive for systems designers who may want to ensure maximal operator compliance. However, this approach may involve risks in the unlikely case that the provided solution is invalid, for example, in cases where vital sensor data is missing or incorrect. Thus, a potential disadvantage of providing operators with augmented feedback or other types of guidance is that operators “blindly” follow the suggested action without checking task-relevant elements of the work domain (Parasuraman et al., 1993). As pointed out by Sheridan (2002), the use of a decision aid implies that the “human can properly decide when the situation includes elements the decision aid can properly assess and can know for which elements the decision aid should be ignored” (p. 150).

The hypothesized risk of decision aids corresponds to theories about “guidance effects” of augmented feedback as studied in the area of motor learning. Wulf and Shea (2004), for example, stated that concurrent augmented feedback “typically has very strong performance-enhancing effects” (p. 128). However, they also noted that, compared to posttrial feedback, concurrent feedback is expected to result in a performance decrement when the feedback is removed. Schmidt and Wulf (1997) argued that concurrent feedback distracts attention from task-intrinsic feedback (Schmidt & Wulf, 1997). Here, intrinsic task feedback is defined as the natural cues in the work environment that are necessary for executing the task correctly, in the absence of augmented feedback.

In the present study, we employed a display called the solution space diagram (SSD; Bijsterbosch et al., 2016). The SSD, which has been used in ATC research, shows the operator whether the current situation is safe or unsafe based on whether the aircraft’s speed vector resides in a no-go zone (a red triangle). In case of a conflict between two aircraft, the operator can reposition the speed vector outside of the no-go zone to resolve a conflict. It is known that ATC operators normally tend to resolve conflicts between aircraft through heading control, whereas speed control seems an underused strategy (Ehrmanntraut, 2004; Hilburn et al., 2014). The SSD shows the operator the entire solution space and therefore facilitates speed control as well as heading control.

Previous research showed that the SSD contributes to reduced self-reported workload during an ATC task as compared to no SSD (Mercado-Velasco et al., 2010). However, it is unknown whether participants who use the SSD may be distracted from processing task-intrinsic cues such as the state of other aircraft shown on the screen. Herein, we used eye-tracking to test the hypothesis of Schmidt and Wulf (1997) that augmented feedback guides attention away from task-intrinsic cues. Thus, besides verifying whether the SSD results in performance improvements (fewer misses and false alarms) and lower self-reported workload as compared to not using the SSD, we examined how participants distributed their visual attention across the display.

## Methods

### Participants

The participants were 24 engineering MSc and PhD students. Their mean age was 24.6 years (*SD* = 4.3 years). The SSD group consisted of 12 males and 1 female and had a mean age of 24.2 years (*SD* = 3.2). The No-SSD group consisted of 10 males and 1 female and had a mean age of 25.0 years (*SD* = 5.2). Participants were allocated in a random manner between the two groups. Ten participants were recruited from the faculty of Aerospace Engineering; the remaining 14 participants were recruited from the faculty of Mechanical Engineering. For the Aerospace Engineering participants, we asked whether the participant was already familiar with the SSD (e.g., from a lecture or research). Two participants who indicated being familiar with the SSD were allocated to the No-SSD group.

This research complied with the American Psychological Association Code of Ethics and was approved by the Human Research Ethics Committee at the Delft University of Technology. Informed consent was obtained from each participant.

### Procedures and Task

First, participants provided their age and gender. Next, they received general instructions, stating:

In this experiment you are asked to perform a *conflict detection task*. You are presented with static Air Traffic Control (ATC) scenarios, each containing two aircraft. For each scenario we need your judgment of whether the two aircraft are on conflicting trajectories, or not. In case the aircraft are in conflict, the aircraft will collide in the future. In case the aircraft are not in conflict, the aircraft will pass by. It is your task to *press the spacebar* if you think the two aircraft are in conflict. In case you think that the aircraft are not in conflict, then do nothing. You are presented with 44 ATC scenarios. Each scenario will last 10 s.

Participants from the No-SSD group and the SSD group were shown a conflict scenario without SSD and the following text:

Here, you see two aircraft represented by square markers. The tip of the black line in front of the marker indicates the future position of the aircraft after one min. This scenario **does contain a conflict**. It is your job to **press the spacebar** when you think the aircraft are in conflict. If you think there is no conflict, **then do nothing**.

This screen was then followed by a screen containing a nonconflict scenario and the following text: “Here another example is given. This scenario does **not** contain a conflict.”

Participants from the SSD group received two extra instruction screens with information about how the SSD worked. First, they were shown the same conflict scenario as before, but now with SSD. The accompanying text said:

In 36 of the trials you are supported by the Solution Space Diagram (SSD). The SSD consists of two circles: The small circle represents the minimum speed of the aircraft (the shortest the speed vector can get); the larger circle indicates the maximum speed of the aircraft (the longest the speed vector can get). The red shape indicates the no-go zone, related to the intruder aircraft. If the tip of the speed vector points into the red triangle, both aircraft are in conflict. **This scenario does contain a conflict**. It is your job to **press the spacebar** when you think the aircraft are in conflict. If you think there is no conflict, **then do nothing**.

On the next screen, participants from the SSD group were shown the same nonconflict scenario as before, now with SSD support. The accompanying text said, “Here another example is given. This scenario does **not** contain a conflict.”

Next, a calibration of the eye tracker was performed, after which the experiment started. The participants then viewed 44 scenarios, each for 10 s. Participants were presented with 36 regular scenarios (3 conflict angles × 2 conflict outcomes, each combination in 6 different configurations) and 8 transfer scenarios (4 conflict angles × 2 conflict outcomes). The transfer scenarios featured no SSD and conflict angles that were different from the conflict angles in the regular scenarios (see Section: Design of the Stimuli). Table 1 provides an overview of the design of the experiment. The order in which the scenarios were presented was identical for every participant.

**Table 1:** Overview of the Scenarios for the Two Experimental Groups

	No-SSD Group	SSD Group
Regular scenarios: 1–18 (11 conflicts, 7 nonconflicts)	No SSD	SSD
Transfer scenarios: 19–22 (4 conflicts, 0 nonconflicts)	No SSD	No SSD
Regular scenarios: 23–40 (7 conflicts, 11 nonconflicts)	No SSD	SSD
Transfer scenarios: 41–44 (0 conflicts, 4 nonconflicts)	No SSD	No SSD

The transfer scenarios were included as an extra feature, with the aim to measure short-term transfer of learning. Because of our limited sample size and limited statistical power, we refrained from a detailed analysis of the transfer trials. Results in this paper are all based on the regular trials; the results regarding the transfer trials can be found in the supplemental material. The transfer results may be useful for defining and designing future research on this topic.

The scenarios all displayed two aircraft on converging tracks. After each scenario, participants rated the difficulty of the preceding trial, by answering the statement “The task was difficult” on a scale of 0 (completely disagree) to 10 (completely agree). The experiment lasted about 15 min per person.

### Apparatus

Eye movements were recorded at 2,000 Hz using the SR-Research Eyelink 1000 Plus. The eye-tracker featured binocular measurements. However, binocular tracking was not always available due to the loss of tracking of one eye. The recorded gaze coordinates of the left and right eye were averaged if left and right were both available.

The stimuli were displayed on a 24-inch BENQ monitor with a resolution of 1,920 × 1,080 pixels (531 × 298 mm). The refresh rate of the monitor was 60 Hz. The distance between the monitor and the head support was approximately 95 cm, and the distance between the eye-tracking camera/IR light source was approximately 65 cm. The monitor suspended a horizontal and a vertical viewing angle of 31° and 18°, respectively.

### Independent Variables

The first independent variable was the availability of the SSD. This was a between-subjects variable. The second independent variable was the conflict outcome. In half of the scenarios, there was a conflict, and in the other half, there was no conflict. In nonconflict scenarios, the distance between aircraft during the closest point of approach (CPA) was 7 nautical miles (NM; 112 pixels or 1.87° on the screen); in conflict scenarios, the CPA was 0 NM. The conflict outcome was a within-subject variable.

### Design of the Stimuli

**Figure 1:**
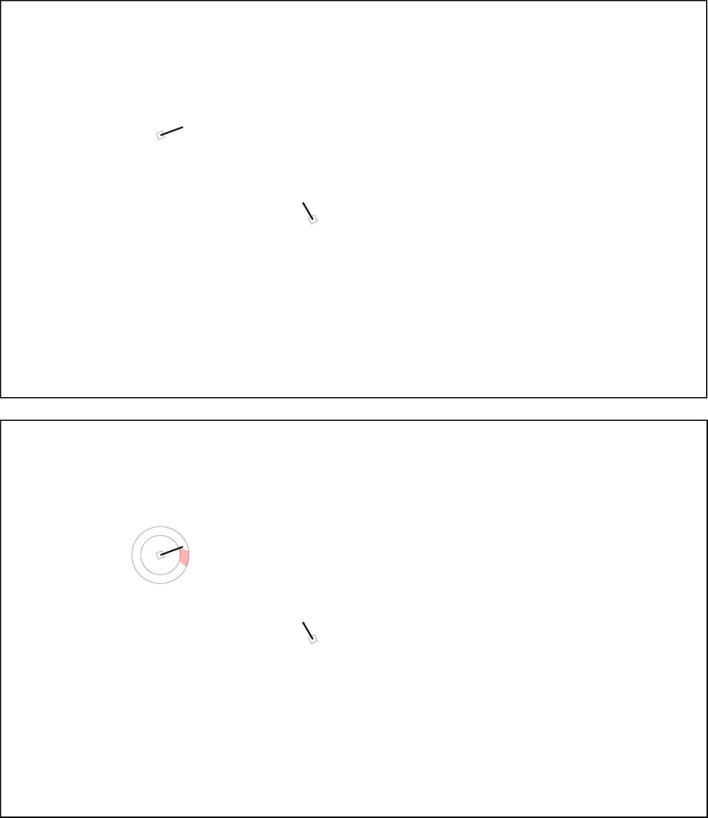
One of the scenarios without conflict (scenario #10). The conflict angle is 100°. Top: no SSD, bottom: SSD. If the tip of the speed vector resides in the red zone, the two aircraft are in conflict. The two concentric circles indicate the minimum and maximum speeds of the aircraft. SSD = solution space diagram.

The scenarios were static ATC images with a resolution of 1,920 × 1,080 pixels. Each scenario featured two aircraft. An aircraft was represented by a square marker with a speed vector (black line) indicating the predicted traveled distance over 1 min, which at a speed of 245 knots corresponds to 4.1 NM or 65 pixels (1.08°) on the screen. Thus, a distance of 1 NM corresponded to 16 pixels (0.27°) on the screen. Figure 1 shows one scenario without and with SSD.

In 22 of the scenarios, the aircraft were in conflict, which meant that a loss of separation would occur after 5 min and that the aircraft would collide. A loss of separation was defined as the moment the distance between the two aircraft dropped below 5 NM (80 pixels, 1.33°). In the other 22 scenarios, the aircraft were not in conflict, which meant that the aircraft safely passed by after 5 min. The closest distance for nonconflict aircraft scenarios was 7 NM (112 pixels, 1.87°). This closest distance of 7 NM was based on pilot tests, where we aimed for an intermediate level of difficulty. That is, we wanted participants to score better than chance (higher than 50% correct performance) but not obtain perfect performance (i.e., lower than 100% correct performance).

Thomas and Wickens (2006) defined three categories of conflict angle between aircraft: (1) overtake: 0°–60°, (2) crossing: 60°–120°, and (3) head-on: 120°–180°. For this experiment, one conflict angle from each of these categories was used. Specifically, we used 30°, 100°, and 150° (12 scenarios per conflict angle). The transfer scenarios had conflict angles of 15°, 35°, 65°, and 145° (two scenarios per conflict angle).

The task was two-dimensional, with the two aircraft flying at the same altitude. The speed of Aircraft 1 (i.e., the aircraft which could potentially contain the SSD) was 245 knots, whereas the speed of Aircraft 2 ranged between 200 and 290 knots. This speed variation between scenarios was implemented to ensure that the scenarios were not perceived as simple geometrical problems. The heading and position of Aircraft 1 (and therefore Aircraft 2) was different for each scenario and obtained using a random number generator. All participants viewed the same 44 scenarios in the same order.

### Dependent Variables

A noncausal median filter with a 100-ms interval was used to cancel out high-frequency camera noise while preserving the information embedded in rapid saccades (see also Eisma, Cabrall et al., 2018). Fixations and saccades were extracted using a standard filter (Eisma, Cabrall et al., 2018). Missing data due to blinks were linearly interpolated. The dependent variables were defined as follows:

- *Self-reported difficulty (0–10)*. A difficulty score between 0 (completely disagree) and 10 (completely agree) was provided by the participants after each scenario.
- *Correct detection (%)*. The percentage of conflict scenarios for which the participant pressed the spacebar.
- *Correct detection response time (RT; ms)*. The mean spacebar response time for conflict scenarios.
- *False positives (%)*. The percentage of nonconflict scenarios for which the participant pressed the spacebar.
- *Mean fixation duration (s)*. During fixations, participants acquire information from the visual array. For calculating the fixation duration, the eye-tracking data were partitioned into saccades and fixations, as in Eisma, Cabrall et al. (2018). First, the gaze speed was filtered with a Savitzky–Golay filter with order 2 and a frame length of 41. A saccade velocity threshold of 2,000 pixels/s was used. The minimum fixation duration was set at 40 ms.
- *Mean saccade amplitude (pixels)*. Saccade amplitude is another common measure in eye-tracking research (Underwood et al., 2011). A higher mean saccade amplitude indicates that participants have a broader spread of fixations on the screen.
- *Gaze coordinates on area of interest (AOI; % of time)*. We computed the percentage of the total fixation time the participants fixated on (1) Aircraft 1 (possibly containing the SSD), (2) Aircraft 2 (never containing an SSD), (3) the conflict point (CP), or (4) along the lines connecting the aircraft and the CP. For Aircraft 1, Aircraft 2, and the CP, a circle of 100-pixel radius (1.67°) was used as a boundary of the AOI. For the connecting lines, a maximum distance to the lines of 50 pixels (0.83°) was used to bound the AOI. The sizes of these AOIs were based on a prior conflict detection task using the same eye tracker (Eisma, Looijestijn et al., 2019). The use of circles of 100-pixel radius ensured sufficient separation of AOIs.

Differences between the SSD and the No-SSD group were compared using independent-samples *t*-tests. An alpha value of .05 was used. The reason for using *t*-tests as opposed to multivariate tests was that we wanted to assess the effect of each dependent variable separately.

## Results

**Figure 2:**
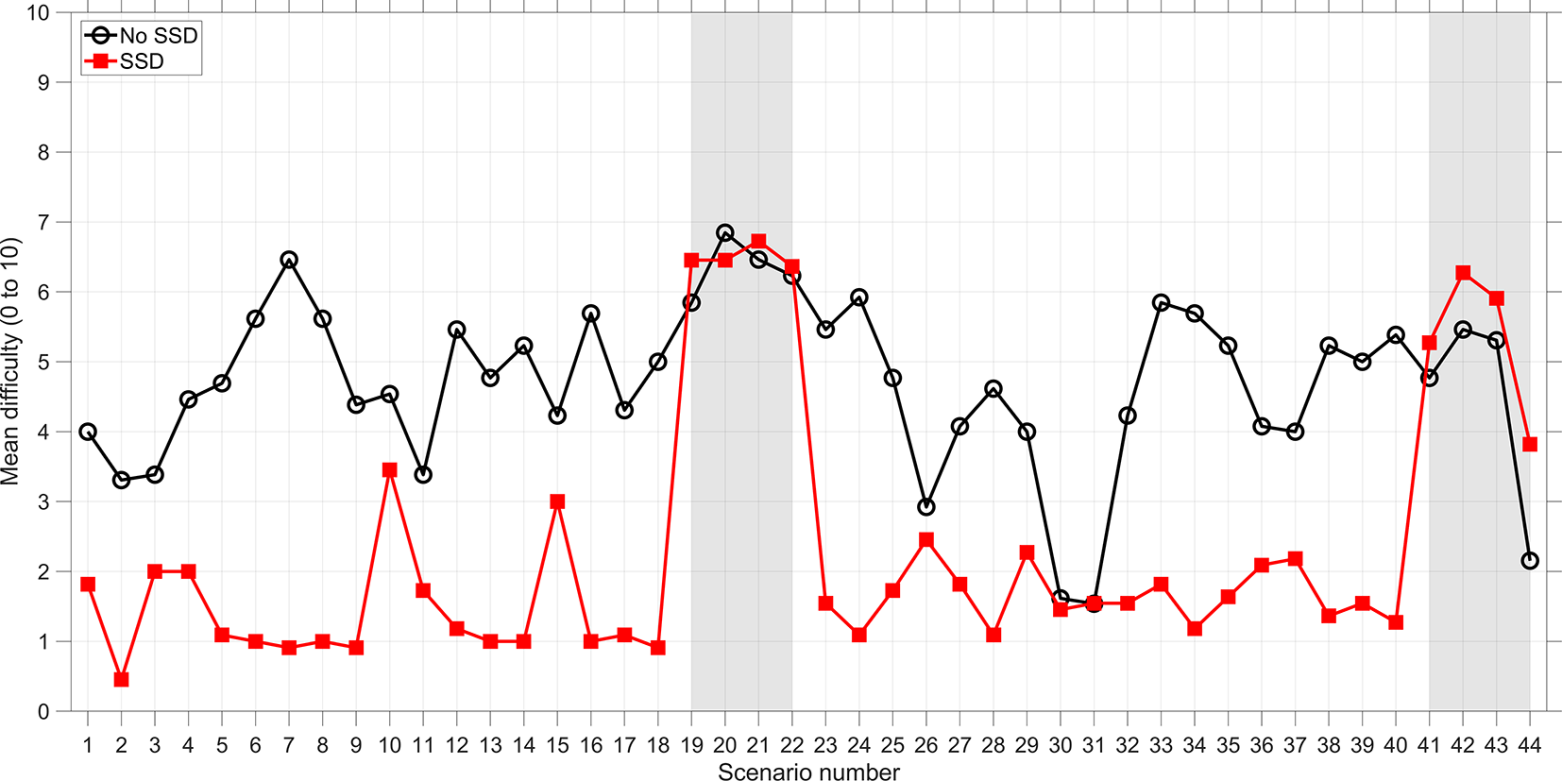
Mean self-reported difficulty as a function of scenario number. Scenarios 19–22 and 41–44 are transfer scenarios. SSD = solution space diagram.

The results in this section are for the regular scenarios (scenarios 1–18, 23–40). The results for the transfer scenarios can be found in the supplemental material. Table 2 shows that participants from the SSD group found the task considerably easier than participants from the No-SSD group. These results are illustrated using Figure 2.

**Table 2:** Means (Standard Deviations in Parentheses) of Dependent Variables for the No-SSD Group and the SSD Group During the Regular Scenarios

	Regular Scenarios			
	No SSD (*n* = 13)	SSD (*n* = 11)	*t*	*p*
Difficulty (0–10)	4.56 (0.94)	1.53 (1.41)	6.26	**<.001**
Correct detection (%)	79.1 (11.1)	93.4 (15.3)	−2.66	**.014**
Correct detection RT (ms)	4,577 (1,285)	2,535 (1,384)	3.75	**.001**
False positive (%)	17.5 (9.8)	14.6 (17.4)	0.51	.617
Saccade amplitude (px)	216 (31)	214 (22)	0.19	.850
Fixation duration (ms)	525 (61)	794 (199)	−4.64	**<.001**
Fixations Aircraft 1 (% of time)	29.4 (5.6)	57.1 (10.4)	−8.27	**<.001**
Fixations Aircraft 2 (% of time)	25.3 (5.9)	13.2 (3.9)	5.83	**<.001**
Fixations CP (% of time)	8.9 (3.9)	5.2 (5.6)	1.90	.070
Fixations lines (% of time)	17.3 (5.1)	9.5 (3.7)	4.20	**<.001**

*Note*. Also shown are the results for independent-samples *t*-tests. Bold formatting indicates p < .05. SSD = solution space diagram

Participants from the SSD group showed a higher conflict detection rate (i.e., more often pressed the spacebar) than participants from the No-SSD group, a statistically significant difference. Participants from the SSD group also detected conflicts significantly faster than the No-SSD participants (Table 2). For nonconflict scenarios, there was no significant difference between the SSD group and the No-SSD group. In other words, the SSD increased correct detections but did not diminish false positives.

**Figure 3:**
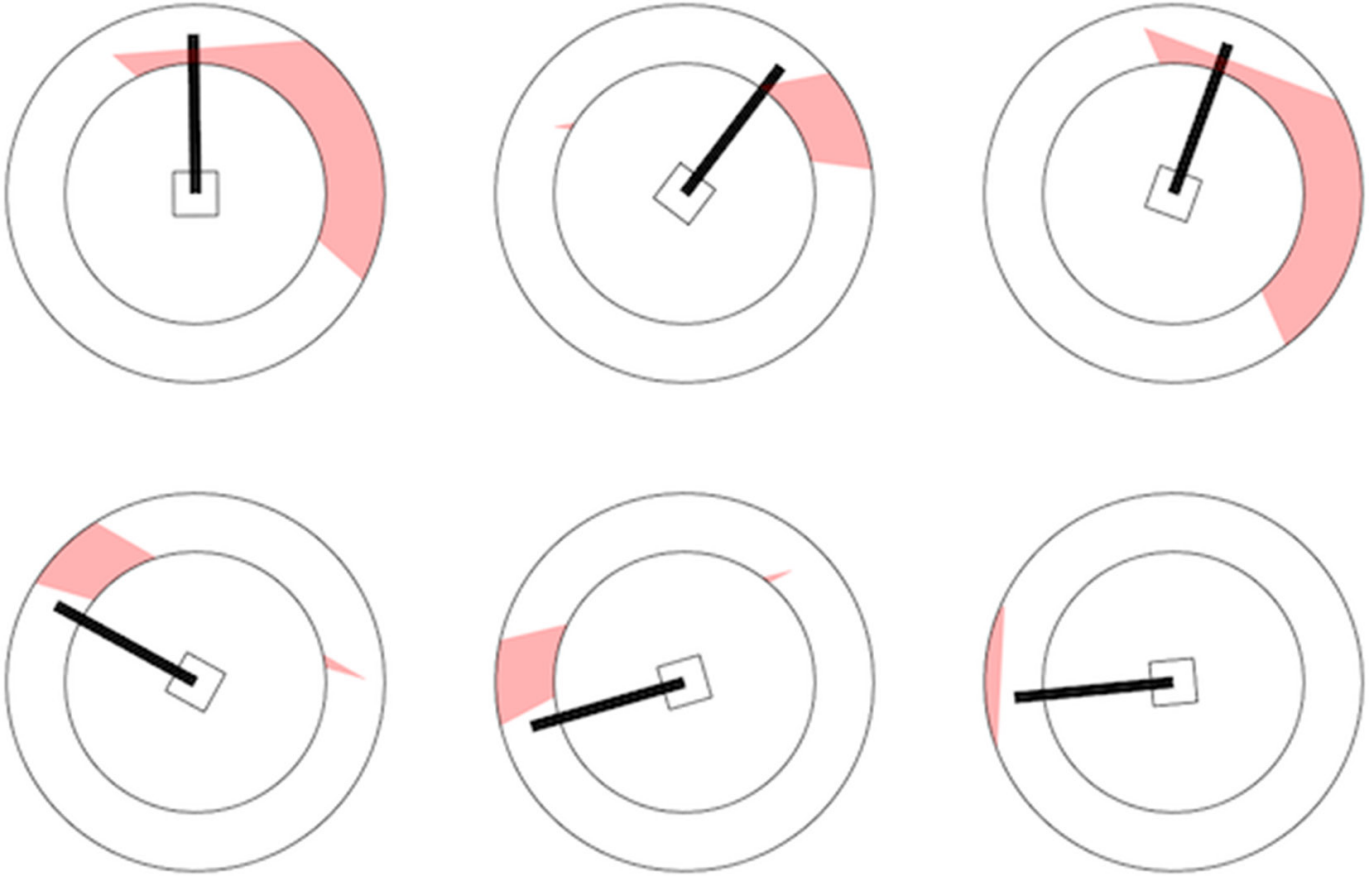
Six selected SSDs in nonconflict scenarios. Top row: SSDs that yielded a high false-positive rate (36%). Bottom row: SSDs that yielded a low false-positive rate (0%). The high false-positive rates may be caused by the fact that the speed vector runs through the red zone. SSD = solution space diagram.

As mentioned above, the SSD did not yield a significantly diminished false-positive rate compared to the No-SSD group, even though the SSD always correctly indicated that the scenario was a no-conflict scenario. To better understand this finding, we explored for which type of scenarios, participants had a high false-positive rate while using the SSD. From the 18 nonconflict scenarios, 6 were of a special kind, where the speed vector ran through the red zone but the tip was in the safe zone. Among the 18 nonconflict scenarios, these 6 scenarios had the highest false-positive rates: 3 scenarios with a false-positive rate of 27% (3 of 11 participants), and 3 scenarios with a false-positive rate of 36% (4 of 11 participants). Figure 3 shows the SSD for the 3 scenarios with a 36% false-positive rate (top row) and 3 scenarios that yielded a false-positive rate of 0% (bottom row). Figure 3 suggests that the high false-positive rates can be explained because participants misunderstood the SSD: The tip is in the safe zone, and hence the aircraft are not in conflict.

**Figure 4:**
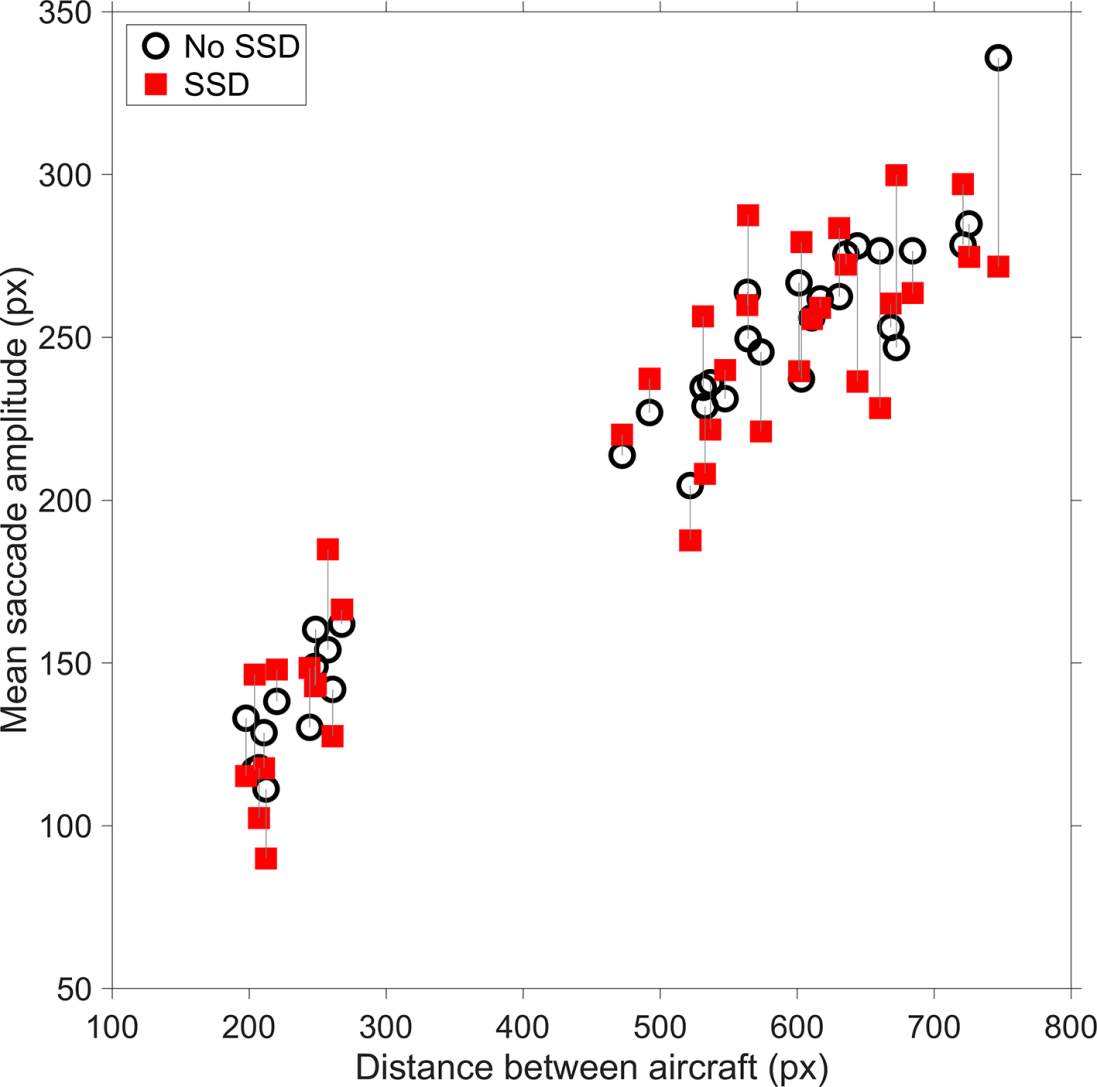
Mean saccade amplitude versus distance between aircraft for the 36 regular scenarios. A vertical line is used to connect the same scenarios. SSD = solution space diagram.

The mean saccade amplitude was not significantly different between the SSD group and the No-SSD group (Table 2). The mean saccade amplitude was strongly dependent on how far the two aircraft were spaced apart (*r* = .97 for no-SSD participants, *r* = .93 for SSD participants, *n* = 44 scenarios, see Figure 4). Thus, the saccade amplitude was scenario-specific and not much influenced by the presence of the SSD.

**Figure 5:**
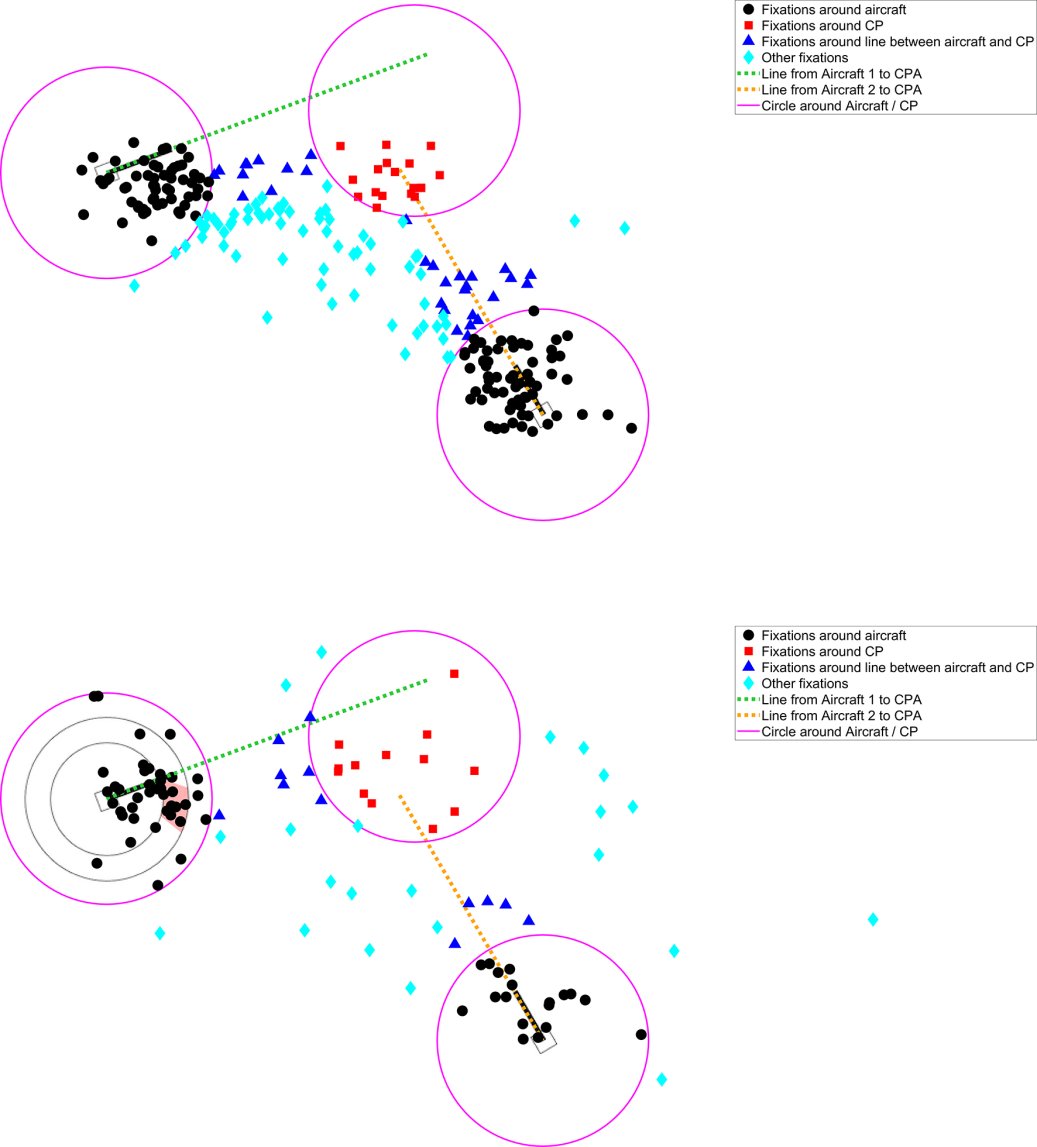
Fixation locations for a nonconflict scenario (scenario #10, see also Figure 1). The top figure shows 237 fixations of 13 participants in the No-SSD group, and the bottom figure shows 108 fixations of 11 participants in the SSD group. Note that the mean fixation duration of participants in the No-SSD group was shorter (466 ms) as compared to participants in the SSD group (1,073 ms). CP = conflict point; CPA = closest point of approach; SSD = solution space diagram..

The participants from the SSD group devoted about twice as much attentional time to Aircraft 1 (which contained the SSD) as compared to participants from the No-SSD group (Table 2). The long viewing durations of the SSD group at Aircraft 1 came at the expense of attention to other areas of interest, in particular Aircraft 2 and the lines between the Aircraft and the CP (Table 2). These findings are illustrated in Figure 5 for one of the scenarios.

**Figure 6:**
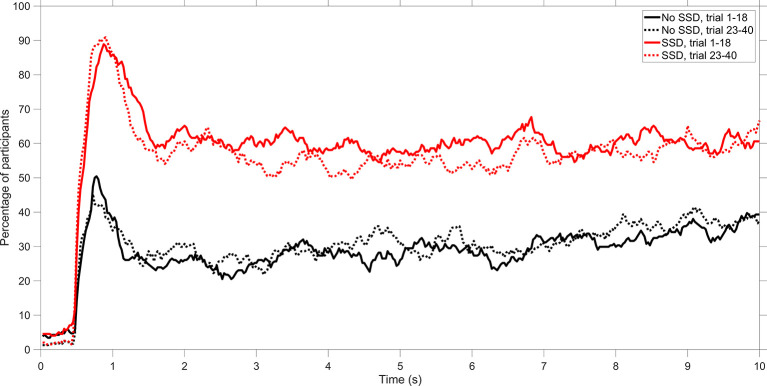
Percentage of participants who looked at the area of interest (AOI) of Aircraft 1. A distinction is made between the No-SSD group and the SSD group and between scenarios 1–18 combined and scenarios 23–40 combined. SSD = solution space diagram.

As a final analysis, we examined the percentage of participants who looked at Aircraft 1 as a function of time during the trial. The results of this analysis, as shown in Figure 6, indicate that Aircraft 1 attracted attention at the start of the trial (i.e., between 0.5 and 1.5 s). Furthermore, no clear learning effects can be distinguished from scenarios 1 through 18 to scenarios 23 through 40.

## Discussion

This study compared self-reported workload, conflict-detection performance, and distribution of visual attention between novice participants who were supported by a visual aid (the SSD) and participants who had to do the task unaided. The results showed that the SSD reduced workload to a substantial extent, from 4.56 to 1.53 on a scale from 0 to 10. Furthermore, with the SSD, participants detected conflicts more accurately and quickly as compared to without the SSD. However, conflict detection with the SSD was imperfect, with a miss rate of 6.6%. There are various possible reasons for this imperfect performance. In particular, participants had only 10 s to respond. Second, it is possible that some participants did not trust the SSD and therefore rejected its indicated correct solution. Disuse is a well-documented phenomenon in the human-automation literature (e.g., Parasuraman & Riley, 1997; Reagan et al., 2019).

The false-positive rates showed no statistically significant differences between the SSD and No-SSD groups. This lack of a significant effect could be due to demand characteristics, where some participants may form a conjecture about the goal of the experiment and adjust their response strategy accordingly. In other words, related to the above explanation about disuse, some participants may have ignored the SSD because they expected that conflicts could still be possible despite the fact the SSD signaled that no conflict was present and was perfectly reliable. Additionally, there are clear indications that some participants misunderstood the SSD. More specifically, some participants did not understand that only the position of the tip of the speed vector is relevant for determining the presence of a conflict. In summary, the SSD was shown to improve conflict-detection performance. However, its effects were not compelling with 6.6% misses and 14.6% false alarms, even though the answer to the conflict-detection task could be readily seen.

We used eye-tracking to measure which elements of the visual scene the participants took into consideration. Results showed that participants from the SSD group allocated more attention to Aircraft 1 (containing the SSD overlay) than participants from the No-SSD group. The attention allocated to the SSD can be interpreted as an epiphenomenon of good task performance or as the cause of good task performance, but also points to dangers in the use of augmented feedback. As augmented feedback comes at the expense of judging the relative positions of relevant aircraft and extrapolating the eye movements toward the CP, collisions may go undetected in (the unlikely) case that the SSD would display incorrect information.

The high amount of attention allocated to the SSD could be because participants needed time to extract information from the SSD; fixation duration is an often-used measure of the difficulty of extracting information (Fitts et al., 1950; Underwood et al., 2011). It could also be that the SSD, because of its salient red color, attracted attention in the absence of other compelling cues in the environment. Besides its appearance, participants themselves may expect the SSD overlay to mean something significant, thereby attracting attention. These notions are consistent with the SEEV model of visual sampling (Wickens & McCarley, 2019), stating that expectancy and visually salient features in the environment are attractors of visual attention.

### Limitations

A limitation of our study is that participants were engineering students, not air traffic controllers. However, this limitation may not have severe consequences because the conflict-detection task was abstract. The “aircraft” flew in a two-dimensional plane, and the stimuli did not feature ATC-specific features such as flight labels. Accordingly, our study measured general perceptual skills, and one should not immediately generalize the findings to ATC applications. Second, the task featured static images, as opposed to dynamic videos or interactive simulations. The use of static images may be realistic for conflict-detection tasks, as regular radar displays should not be expected to have a high update rate. Third, our study was concerned with conflict detection only. The SSD also facilitates opportunities for conflict resolution, something that was not studied herein. However, we argue that, based on Parasuraman et al.’s (2000) stages of information processing, conflict detection necessarily precedes conflict resolution; it is not possible to resolve a conflict if that conflict is not detected first. Fourth, although the SSD consists of nothing more than two circles, a red polygon, and a vector, it was still misunderstood by a number of participants. Future research could use even simpler displays, such as a salient warning signal or a text message as used in traffic collision avoidance systems (e.g., “traffic, traffic”). It can be expected that simpler displays reduce the visual load but are also more prone to guidance effects. Winstein et al. (1994) hypothesized that “feedback that is relatively more guiding would be expected to have greater detrimental effects on motor learning” (p. 317).

### Recommendations and Implications

The question may arise as to whether augmented displays like the SSD represent what they intend to represent. Borst et al. (2019) stated that the SSD “portrays velocity obstacles (or, conflict zones) in speed and heading within the maneuvering envelope of the aircraft under control” (p. 624). An important question is whether people indeed see “velocity obstacles” and not merely “lines and a red shape” without further understanding of the work domain. Future research could use interviews, self-reports, or think-aloud methods to examine what people are phenomenologically perceiving. Furthermore, the perceptual task that was used in our study may not exploit the SSD to its fullest potential. Future research could apply augmented feedback in complex supervisory tasks, where knowledge development is important.

Our work has several implications for display design. Intuitively, it may be expected that display augmentation, whether it be the SSD or any other type of additional visual information, improves performance (Maddox, 1996). Our study showed that augmented feedback from the SSD did improve performance, with the correct detection rate increasing from 79.1% to 93.4% and the false-positive rate decreasing from 17.5% to 14.6%. These improvements may be regarded as underwhelming because the SSD always showed the correct solution, and 100% accuracy should therefore be possible. Clearly, the SSD is no panacea, and participants require more instructions or training about how to use the SSD; such extended training/instructions may be expected to reduce the participants’ error rates caused by the confusing SSD design and may facilitate proper reliance on the SSD. It was also shown that augmented feedback attracts attention at the expense of other elements in the environment at no cost to performance. Finally, the SSD was misunderstood in some scenarios. This finding may have been preventable by providing participants with more explicit instructions about how to interpret the SSD. At the same time, this finding serves as a caution for HMI designers, as it shows that augmented feedback that is designed to increase task performance can actually reduce task performance. Our observations are in line with Yeh et al. (2003), who concluded that extraneous visual elements hinder target detection.

Our findings demonstrate that augmented feedback that is intended to improve conflict-detection performance has side effects in the form of attentional demands and misunderstanding. Accordingly, we recommend that augmented feedback should be used with appropriate caution. Better options might be to offer a more explicit form of decision support that uses minimal visual clutter or to fully automate the decision-making task if the automation is sufficiently reliable.

## Key Points

- The effect of visual augmented feedback was studied in a conflict-detection task.
- Results show improved hit rate but no improved false-positive rate compared to baseline.
- Some false positives are attributed to operator misunderstanding of the augmented feedback.
- Eye-tracking results show that augmented feedback attracts visual attention.

## Supplemental Material

Supplementary data - Supplemental material for Augmented Visual Feedback: Cure or Distraction? Click here for additional data file. Supplemental material, Supplementary data, for Augmented Visual Feedback: Cure or Distraction? by Yke Bauke Eisma, Clark Borst, René van Paassen and Joost de Winter in Human Factors: The Journal of Human Factors and Ergonomics Society
