# Supplementary material for: Augmented Visual Feedback: Cure or Distraction?
Source: Hum Factors. 2020 Jun 3;63(7):1156–68. doi: 10.1177/0018720820924602 (PMC8521352; doi:10.1177/0018720820924602)
Supplement: Supplementary data - Supplemental material for Augmented Visual Feedback: Cure or Distraction? [file sj-docx-1-hfs-10.1177_0018720820924602.docx]

**Supplementary Materials: Analysis of Transfer Trials**

We included a number of transfer scenarios without the SSD to examine guidance or training effects. Here, guidance effects would be confirmed if participants who previously used the SSD perform *worse* than participants who have never used the SSD, and training effects would be confirmed if participants who previously used the SSD perform *better* than those who never used the SSD.

Scenarios 19–22 contained no conflict, and Scenarios 41–44 contained a conflict. This design allowed us to inspect whether transfer effects faded out with scenario number, that is, whether the transfer effect was stronger during the first transfer scenario (i.e., Scenario 19 for conflicts, and Scenario 41 for non-conflicts) compared to the fourth transfer scenario (i.e., Scenario 22 for conflicts and Scenario 44 for non-conflicts).

During the transfer scenarios, where the participants of the SSD group had to do the task without SSD (scenarios 19–22, 41–44), self-reported difficulty increased back to levels equivalent to the No-SSD group (Table S1 & Figure 2). There were again no significant differences between the two groups for any of the dependent variables (Table S1).

Table S1.

*Means (standard deviations in parentheses) of dependent variables for the No-SSD group and the SSD group during the transfer scenarios. Also shown are the results for independent-samples t-tests.*

|  | **Transfer scenarios** | | | |
| --- | --- | --- | --- | --- |
|  | **No SSD (*n* = 13)** | **SSD (*n* = 11)** | ***t*** | ***p*** |
| Difficulty (0 to 10) | 5.38 (1.13) | 5.91 (1.59) | -0.94 | .356 |
| Correct detection (%) | 63.5 (16.5) | 68.2 (16.2) | -0.70 | .488 |
| Detection RT (ms) | 5981 (1646) | 6268 (1007) | -0.50 | .619 |
| False positive (%) | 9.6 (19.2) | 20.5 (15.1) | -1.52 | .144 |
| Saccade amplitude (px) | 181 (34) | 168 (22) | 1.16 | .258 |
| Fixation duration (ms) | 546 (59) | 574 (100) | -0.85 | .407 |
| Fixations Aircraft 1 (% of time) | 21.4 (5.6) | 19.0 (8.3) | 0.87 | .395 |
| Fixations Aircraft 2 (% of time) | 39.8 (7.2) | 33.0 (9.5) | 2.00 | .058 |
| Fixations CP (% of time) | 7.1 (4.5) | 10.7 (7.3) | -1.45 | .162 |
| Fixations lines (% of time) | 14.7 (8.7) | 16.9 (6.8) | -0.68 | .505 |

Summarizing, our study found no significant transfer effects (Table 2) nor visible experience effects during the regular scenarios (Figures 2 & 6). We showed that when the SSD was withdrawn, participants dropped back to unaided levels of performance and workload. Accordingly, we did not confirm the guidance hypothesis; participants from the SSD group did not perform significantly worse than participants from the No-SSD group in transfer (i.e., there was no ‘negative transfer’). However, our experiment also did not find any evidence for the suitability of SSD as a training tool, as no positive transfer effect was identified. In other words, in the context of our conflict-detection task, the training value of the SSD is debatable. It must be noted that we did not use the SSD as part of a training program that is designed to maximize transfer-of-learning. Such a training program could consist of a scaffolding approach by gradually decreasing the amount of information shown by the SSD (Beed, Hawkins, & Roller, 1991). Thus, the present results reflect the ‘plain’ learning value of the SSD, not its potential learning value within a dedicated learning environment.

It should be noted that with our sample size of 24, we had limited statistical power. Using G*Power software (Faul et al., 2007), we computed the required effect size for a two-group research design (*n_1_* = 13, *n_2_* = 11), assuming a false positive rate of 5% and a statistical power of 80%. The results of this analysis showed that the required effect size (Cohen’s *d*) is 1.20, a very strong effect. Hence, our design was powerful enough for detecting strong differences while the SSD was present (see Table 2), but not powerful enough for detecting any small transfer effects that may exist. The lower power can be illustrated using Table S1, where a substantial difference in false positives is depicted (20.5% with SSD, 9.6% without SSD) while this effect is not significant (*p* = 0.058). Note that a Wilcoxon test for the false positives also yielded no significant effect between the SSD and no-SSD groups (*p* = 0.065). An issue here is that there were only four non-conflict transfer scenarios (Scenarios 41–44), and therefore the false-positive rate for a participant could be either 0%, 25%, 50%, 75%, or 100%, resulting in high variance between participants. Statistical power is expected to increase when using more participants and more scenarios per participant. For future research into transfer-of-training, we recommend using larger sample sizes.

Another explanation for the lack of transfer effects may lie in the duration of the experiment. The experiment may be too short for any substantial learning effect to occur, as learning within our experimental context (ATC) is a process that often takes days or even weeks.

Our findings are consistent with Borst et al. (2019), who found no statistically significant differences in conflict detection performance during transfer scenarios between participants who had completed a two-day training program using an SSD and participants who had received only instructions. Similarly, Van Leeuwen, De Groot, Happee, and De Winter (2011) found that, in a driving simulator, continuous visual feedback on the lateral position enhanced lane-keeping performance, yet attracted substantial amounts of visual attention and did not yield significant differences with a control group in a retention trial.

In the present study, we used transfer scenarios without SSD; future research could examine cases in which the SSD provides erroneous information, as previously explored by Bijsterbosch et al. (2016). Also, future research could examine whether the SSD benefits the learning of the essentials of conflict detection or whether it supports overreliance that inhibits performance when the SSD is removed. Instead of measuring only eye movements and conflict detection performance, future research could employ interviews, knowledge tests, of think-aloud methods to shed light on participants’ cognitive processes.
